# Germline Variants in the POT1-Gene in High-Risk Melanoma Patients in Austria

**DOI:** 10.1534/g3.117.300394

**Published:** 2018-03-09

**Authors:** Christoph Müller, Milica Krunic, Judith Wendt, Arndt von Haeseler, Ichiro Okamoto

**Affiliations:** * Department of Dermatology, Medical University of Vienna, Austria; † Center for Integrative Bioinformatics Vienna, Max F. Perutz Laboratories, University of Vienna, Medical University Vienna, Austria; ‡ Bioinformatics and Computational Biology, Faculty of Computer Science, University of Vienna, Austria

**Keywords:** POT1, melanoma, familial, genetics, Austria

## Abstract

Risk of melanoma is in part determined by genetic factors. Currently the only established high penetrance familial melanoma genes are CDKN2A and CDK4. Recent studies reported germline variants in POT1 in melanoma families. In the present study, we sequenced the entire POT1 gene in 694 patients from the M3-study. Patients with multiple primary melanomas (n = 163) or with a positive family history (n = 133) were classified as high-risk melanoma patients. Additionally, 200 single primary melanoma patients and 198 non-melanoma controls were sequenced. For prediction analysis 10 different tools were used.

In total 53 different variants were found, of which 8 were detected in high-risk melanoma patients, only. Two out of these 8 variants were located in exons and were non-synonymous: g.124510982 G>A (p.R80C) and g.124491977 T>G (p.N300H). While g.124491977 T>G was predicted to be neutral, 80% of the prediction tools classified g.124510982 G>A as deleterious. The variant, g.124467236 T>C, which possibly causes a change in the splice site was identified in a case with a positive family history in the present study. Another variant in the 5-UTR, g.124537261 A>G, was found in 2 high-risk patients. So, in conclusion, melanoma associated POT1 germline variants seem to be rare. Further studies are required to evaluate the role of POT1 for genetic counseling.

Approximately 10–15% of all melanoma patients report a positive family history, multiple primary melanomas or early onset of melanoma diagnosis (Müller *et al.* 2016). The most important high penetrance gene is the cyclin-dependent kinase Inhibitor 2A (CDKN2A), responsible for about 30% of all familial melanoma cases. Melanoma associated mutations in cyclin-dependent kinase 4 (CDK4), which were also classified as high penetrance mutations, seem to be very rare as only a few families were reported since the initial report in 1996 (zuo *et al.* 1996). Only recently, a mutation in the telomerase reverse transcriptase gene (TERT) was described in melanoma patients, adding further data to the already existing evidence that stability of telomeres is important in melanoma biology.

Shelterin, a protein complex composed of six subunits, is involved in the protection of the chromosome ends and in the regulation of the telomerase activity (aoude *et al.* 2015). Recently this complex gained particular interest in melanoma genetics as germline variants were found in 3 shelterin genes in melanoma prone families (robles-espinoza *et al.* 2014; shi *et al.* 2014; aoude *et al.* 2015): POT1, ACD and TERF2IP. The human POT1 gene is located at 7q31.33 and has 19 transcripts. The isoform 1 of the protein, where the variants were originally found, consists of 19 exons and of 634 amino acids. Since the initial description of POT1 as a predisposition gene for hereditary melanoma (robles-espinoza *et al.* 2014; shi *et al.* 2014), no further variants associated with melanoma has been described except for one in a single melanoma prone family in the U.S.A. (wilson *et al.* 2017). Therefore, the frequency of these variants in other populations remains unclear. This information is crucial to decide whether high-risk patients should be tested for POT1 in a routine genetic counseling of melanoma families (goldstein *et al.* 2007). Here we present for the first time data of POT1 variants in high-risk melanoma patients in Austria.

## Patients and Methods

### Study participants

In total, DNA of 694 participants was analyzed. All participants were Caucasians with European ancestry and were recruited in Austria as described elsewhere (burgstaller-muehlbacher *et al.* 2015). High-risk melanoma patients (n = 296), included patients with multiple primary melanomas (n = 163) and patients with a positive family history (n = 133) and were compared to a reference group of single melanoma (n = 200) and non melanoma patients (n = 198). Descriptive data were shown for gender, age at diagnosis, Breslow index, tumor localization and histological subtype in Table 1. In multiple primary melanoma patients, data (date of surgery, localization, histological description such as histological subtype and Breslow index) refers to the first primary melanoma. Informed consent was obtained from all individual participants included in the study. The study was approved by the ethics committee of the Medical University of Vienna.

**Table 1:** Participant characteristics

		Controls	SPM	PFH	>1 PM
Gender	female	74	80	60	55
	male	124	120	73	108
Mean age (SD)		53.8 (15.8)	52.6 (16.4)	49.7 (15.9)	53.9 (15.1)
	Missing	0	2	1	0
Mean Breslow in mm (SD)		—	1.4 (1.9)	1.1 (1.3)	1.1 (1.3)
	Missing/Occult		10/4	4/3	9/0
Localization	Head and Neck	—	15	16	24
	Upper Extremity	—	21	12	13
	Trunk	—	115	72	85
	Lower Extremity	—	43	30	40
	Missing/Occult	—	2/4	0/3	1/0
Histological Subtype	LM/LMM	—	8	9	19
	NMM	—	38	19	25
	SSM	—	87	74	74
	others	—	67	31	45

SD: standard deviation; LM: lentigo maligna; LMM: lentigo maligna melanoma; NMM: nodular melanoma; SSM: superficial spreading melanoma; SPM: single primary melanoma; PFH: patients with a positive family history; >1 PM: patients with multiple primary melanomas.

### Genotyping

The DNA was purified from whole blood as described previously (burgstaller-muehlbacher *et al.* 2015). Next generation-sequencing of POT1 was performed at the Genome Centre, Queen Mary, University of London (http://www.smd.qmul.ac.uk/gc/). For the preparation of DNA libraries 0.5 μg of genomic DNA was used. Amplicon libraries were created with the Fluidigm Access Array according to the manufacturer’s protocol. The 150-bp paired-end sequencing was done on the Illumina MiSeq v2 platform.

The datasets generated during the current study are available in the NCBI Sequence Read Archive (https://www.ncbi.nlm.nih.gov/sra).

### Data analysis

The reads were mapped against human genome reference (hg19) using NextGenMap (sedlazeck *et al.* 2013) (v0.5.0) with default parameters plus several additional options: identity (-i) was set to 0.85, maximum number of consecutive indels allowed (-C) was set to 120 and we used alignment algorithms that support affine gap costs (–affine). Read groups in aligned reads (BAM files) were replaced using Picard tools (http://broadinstitute.github.io/picard) option AddorReplaceReadGroups. The aligned reads were then indexed using SAMtools (li *et al.* 2009) (v1.1). Local realignment around insertions and deletions and quality base score recalibration were performed using the Genome Analysis Tool Kit (mckenna *et al.* 2010) (GATK, v2.6). To call variants (SNPs and indels) in aligned reads files, we used UnifiedGenotyper from GATK with parameters: -dcov set to 2000,–standard_min_confidence_threshold_for_calling set to 30.0, - standard_min_confidence_threshold_for_emitting set to 10, -glm set to BOTH and for option–dbsnp we used human_9606 variants from dbSNP database (sherry *et al.* 2001). GATK called variants were first divided into SNPs and indels using SelectVariant. SNPs to be filtered out were labeled using VariantFiltration with the following filter expressions:–clusterWindowSize =10, “MQ0 >= 4 && ((MQ0 / (1.0 * DP)) > 0.1)”, “DP < 5”, “QUAL < 30.0 QUAL > 30.0 && QUAL < 50.0”, “QD < 0.8”, “FS > 60.0”and

–missingValuesInExpressionsShouldEvaluateAsFailing. Indels to be filtered out were labeled using VariantFiltration with the filter expressions: “QD < 2.5 || ReadPosRankSum < -20.0 || FS > 200.0”,

“–missingValuesInExpressionsShouldEvaluateAsFailing”. The variants were then combined by GATK CombineVariants tool. Rearranging results was done using our in-house developed python and R scripts.

### Prediction analysis of non-synonymous POT1 variants

Two non-synonymous POT1 variants, found in high risk melanoma patients only, were analyzed using 10 prediction tools as described previously (burgstaller-muehlbacher *et al.* 2015; Müller *et al.* 2016): MutationTaster2 (schwarz *et al.* 2014), PolyPhen-2 (Polymorphism Phenotyping-v2, HumDiv and HumVar) (adzhubei *et al.* 2010), PROVEAN (Protein Variation Effect Analyzer) (choi *et al.* 2012), SIFT (sorts intolerant from tolerant substitutions) (ng and henikoff 2001), SNAP2 (screening for non-acceptable polymorphisms-2) (bromberg and rost 2007), PANTHER (Protein ANalysis THrough Evolutionary Relationships) (mi *et al.* 2013), CADD (Combined Annotation Dependent Depletion) (kircher *et al.* 2014), GERP++ (davydov *et al.* 2010) and phyloP (pollard *et al.* 2010). For the latter 2, the tables of the UCSC genome browser tb_allHg19RS_BW and phyloP46wayPlacental were used. Most of those tools provide information about the effect of an amino acid exchange on the protein function. GERP++ and phyloP give a score depending on the conservation by comparing different species. The cut-off score for PROVEAN was -2.5, values below indicate the prediction as deleterious. In SIFT, values have a range from 0 to 1, whereas a score below 0.05 means that the variant is predicted to be deleterious. In CADD values above 15 were classified as deleterious. The range Polyphen2 scores is from values of 0 to 1; higher scores are more likely to be found in deleterious variants with a cut-off score of 0.5. SNAP 2 has output scores between -100 (strong neutral prediction) to 100 (strong effect prediction). PANTHER calculates the preservation time to give a prediction. Longer times indicate a more likely functional impact.

As protein sequence for the data input, the POT1 isoform 1 (ENST00000357628) was used.

### Data availability

All raw sequencing data are deposited in the NCBI Sequence Read Archiv (SRA) under the BioProject ID PRJNA400454.

## Results

### POT1 variants in the entire study population

Descriptive data of the study population is shown in Table 1. In 694 sequenced individuals, we found 53 genetic variants, 21 of which were not listed in the dbSNP (sherry *et al.* 2001) (see Table 2); 48 were detected in melanoma patients exclusively and 5 additional variants in the control group only. Out of 53 variants, 27 were located in introns, 7 in the 5′ untranslated region (UTR), 10 in the 3′ UTR and 9 in exons (see Table 2). Of the latter, 8 resulted in an amino acid exchange and 1 was synonymous. Three non-synonymous variants were located at exon 9, 2 at exon 11 and 1 at exon 7, 14 and 17, respectively. The most common variants in the exons were p.G404V (21 participants), followed by p.D185E (4 participants) and p.V183G (3 participants). All 3 variants were found in cases as well as controls and were listed in the dbSNP (sherry *et al.* 2001).

**Table 2:** All variants with localization and their distribution

Localization	Position	dbSNP	Aminoacid exchange	REF	ALT	Controls	SPM	PFH	>1 PM
5′UTR	124503574	n.a.	—	T	C	0	1	0	0
5′UTR	124537261	rs202009081	—	A	G	0	0	1	1
5′UTR	124568913	n.a.	—	C	T	0	0	0	1
5′UTR	124568914	rs535705635	—	G	A	0	2	0	0
5′UTR	124568963	rs118121031	—	T	A	3	1	2	3
5′UTR	124569916	rs117811540	—	G	A	3	3	4	4
5′UTR	124569930	rs568780254	—	C	T	2	1	3	1
Intron	124465256	rs146966778	—	T	C	2	3	3	5
Intron	124465509	rs10250202	—	A	C	131	118	95	110
Intron	124467236	rs749702835	—	T	C	0	0	1	0
Intron	124469267	rs10263573	—	A	T	131	118	95	110
Intron	124469495	n.a.	—	AATT	A	0	1	0	0
Intron	124475296	rs66826272	—	TAAACA	T	74	76	65	75
Intron	124475296	rs369649621	—	T	TAAACA	44	63	0	33
Intron	124477182	rs7787804	—	A	G	179	183	122	149
Intron	124477188	n.a.	—	T	C	1	0	0	0
Intron	124477270	rs144116156	—	A	G	0	2	1	0
Intron	124481245	rs3815221	—	G	A	131	118	94	110
Intron	124482746	n.a.	—	AAATAT	A	0	1	0	0
Intron	124486898	n.a.	—	T	C	1	0	0	0
Intron	124486928	n.a.	—	G	C	1	0	0	1
Intron	124486968	n.a.	—	C	T	1	0	1	0
Intron	124486980	rs7794637	—	T	C	179	183	122	149
Intron	124486985	n.a.	—	CAT	C	1	0	0	0
Intron	124487064	n.a.	—	A	AAAAGGC	0	1	0	0
Intron	124491886	rs182906205	—	T	C	1	0	0	0
Intron	124492038	rs7784168	—	T	C	88	108	63	90
Intron	124492970	rs751428333	—	T	C	0	1	0	0
Intron	124499002	rs6977407	—	A	C	161	176	101	138
Intron	124499003	rs6959712	—	T	A	161	176	101	138
Intron	124537283	rs112411545	—	A	G	2	1	2	0
Intron	124538285	rs10229152	—	G	A	162	176	100	139
Intron	124538436	rs57468586	—	GA	G	168	164	107	121
Intron	124555710	n.a.	—	G	GA	0	1	0	0
Exon 7	124510982	rs778692211	p.R80C	G	A	0	0	0	1
Exon 9	124499165	rs200464979	p.V183G	A	C	1	1	1	0
Exon 9	124499158	rs750899684	p.D185E	A	T	2	0	1	1
Exon 9	124499092	n.a.	p.L207F	T	A	1	0	0	0
Exon 11	124491951	rs34398311	p.Q308=	T	C	0	1	0	0
Exon 11	124491972	rs116916706	p.Q301H	C	A	0	1	0	0
Exon 11	124491977	n.a.	p.N300H	T	G	0	0	0	1
Exon 14	124481185	rs35536751	p.G404V	C	A	9	8	3	1
Exon 17	124467270	n.a.	p.S562P	A	G	0	1	0	0
3′UTR	124462448	n.a.	—	A	C	0	1	0	0
3′UTR	124462617	rs544668410	—	A	C	0	2	0	1
3′UTR	124462655	rs76436625	—	T	C	38	42	28	36
3′UTR	124462661	rs17246404	—	C	T	100	108	53	75
3′UTR	124463018	rs530211997	—	C	T	0	0	0	1
3′UTR	124463391	n.a.	—	CTA	C	159	179	114	135
3′UTR	124463400	n.a.	—	T	C	0	0	0	1
3′UTR	124463428	rs142378997	—	T	G	4	4	5	2
3′UTR	124463559	n.a.	—	T	C	0	0	0	1
3′UTR	124463612	n.a.	—	T	C	0	1	0	0

n.a.: not available; REF: reference sequence; ALT: alteration; SPM: single primary melanoma; PFH: patients with a positive family history; >1 PM: patients with multiple primary melanomas.

### POT1 variants in high-risk patients

Eight variants were exclusively found in high-risk melanoma patients (see Table 3). Four of these 8 variants were not listed in the dbSNP. Of all variants detected in high-risk melanoma patients exclusively (n = 8), 2 were located in the 5′UTR, 3 in the 3′ UTR, 1 in an intron and 2 in exons; 1 in exon 7 and another in exon 11. The latter 2 (g.124510982 G>A and g.124491977 T>G) were both found in one multiple primary melanoma patient each. The carrier of g.124510982 G>A, was a male patient, diagnosed with an amelanotic melanoma at the age of 33 with a second melanoma excised 35 years later and was tested wild type for CDKN2A. The carrier of the other non-synonymous variant, g.124491977 T>G, was 57 years old when his first primary melanoma was excised. Nine years later, an in-situ melanoma was found on his back.

**Table 3:** High risk patients and melanoma characteristics

Variant	dbSNP	Carrier	No. of primaries	1^st^ melanoma	2^nd^ melanoma	3^rd^ melanoma	4^th^ melanoma	CDKN2A status	Family history of melanoma
				Age/Breslow/Localization	Age/Breslow/Localization	Age/Breslow/Localization	Age/Breslow/Localization		
g.124537261 A>G	rs202009081	PFH	1	49/0.4mm/Lower Extremity	—	—	—	wt	Mother 68 years
g.124537261 A>G	rs202009081	>1 PM	4	47/0.3mm/Shoulder	70/*in situ*/Lower extremity	71/*in situ*/Back	74/2.4mm/Back	wt	negative
g.124568913 C>T	n.a.	>1 PM	2	66/1mm/Lower extremity	74/5mm/Genital	—	—	wt	negative
g.124467236 T>C	rs749702835	PFH	1	22/0.4mm/Abdomen	—	—	—	c.151-4 G>GC	Mother 40 years
g.124510982 G>A	rs778692211	>1 PM	2	33/Unknown/Lower extremity	68/0.45mm/Back	—	—	wt	negative
g.124491977 T>G	n.a.	>1 PM	2	57/1mm/Back	66/*in situ*/Back	—	—	wt	negative
g.124463018 C>T	rs530211997	>1 PM	3	44/0.75mm/Chest	53/1.6mm/Back	57/0.4mm/Chest	—	wt	negative
g.124463400 T>C	n.a.	>1 PM	2	31/0.5mm/Chest	31/*in situ*/Lower extremity	—	—	wt	negative
g.124463559 T>C	n.a.	>1 PM	2	36/1mm/Abdomen	53/*in situ*/Lower extremity	—	—	p.R24P	negative

n.a.: not available; PFH: patients with a positive family history; >1 PM: patients with multiple primary melanomas, wt: wild type.

Of the variants listed in the public SNP databases, g.124467236 T>C, which was described in a patient with multiple primary melanomas before (shi *et al.* 2014), was found in our study in a female patient with a positive family history. She was diagnosed at the age of 22 while her mother had her diagnosis at the age of 40 (which could be confirmed by medical records), conforming with the criteria for inherited risk of melanoma.

The variant g.124537261 A>G located in the 5′UTR, was the only one found in 2 high-risk patients. Both were diagnosed for melanoma before the age of 50 and tested wild type for CDKN2A mutations. One had a positive family history for melanoma and the other patient was diagnosed with 4 primary melanomas.

Two variants located in the 3′UTR, g.124463018 C>T and g.124463400 T>C, were found in early onset patients with multiple primary melanomas each, both tested wild type for CDKN2A mutations.

### Prediction analysis of non-synonymous POT1 variants

Prediction analysis was performed for non-synonymous variants in coding sequences which were only found in high-risk melanoma patients: g.124491977 T>G and g.124510982 G>A, respectively. While g.124491977 T>G was predicted to be neutral by all of the used prediction tools, the variant g.124510982 G>A was predicted to be deleterious by 8 of 10 prediction tools (80%). Results of all prediction analyses are shown in Table 4.

**Table 4:** Prediction of the variant g.124510982 G>A and g.124491977 T>G

Prediction tools		g.124510982 G>A	g.124491977 T>G
MutationTaster	Prediction	Disease causing	Polymorphism
Polyphen2	HumDiv	Probably Damaging	Benign
	Score	0.987	0.168
	HumVar	Possibly Damaging	Benign
	Score	0.791	0.048
Provean	Prediction	Deleterious	Neutral
	Score	−5.503	−0.623
Sift	Effect	Tolerated	Tolerated
	Score	0.16	0.11
CADD	PHRED 12 score	31	0.014
SNAP2	Prediction	Neutral	Neutral
	Score	−15	−89
	Expected acc.	57%	93%
Panther	Preservation time	1628	91
	Message	Probably damaging	Probably benign
GERP++	Score	5.57	−4.9
PhylOP	Score	2.77	−0.470331
Sum deleterious	Total	8	0
	in %	80	0

### Coincidence of CDKN2A mutations

To exclude coincidence with CDKN2A mutations, we then examined the CDKN2A sequence of our cases carrying potential risk variants of POT1. One of the variants found exclusively in high-risk patients, g.124463559 T>C, was associated with an established CDKN2A high-risk mutation, g.21974756 C>G (p.R24P). The carrier of the POT1 variant, g.124467236 T>C, additionally had the CDKN2A variant g. 21971211 G>C (c.151-4 G>GC), which was demonstrated to be non-effective in a previous study (burgstaller-muehlbacher *et al.* 2015).

## Discussion

Only recently, novel disease associated germline variants in POT1 were reported in melanoma pedigrees (robles-espinoza *et al.* 2014; shi *et al.* 2014). This finding is of particular interest as the established disease causing mutations in familial melanoma, *i.e.*, mutations in CDKN2A and CDK4 account only for 30–40% of the melanoma pedigrees. Despite this, just one family with a POT1 germline variant associated with melanoma was published so far (wilson *et al.* 2017).

In the present study, in which the entire POT1 gene was sequenced in cases at high risk of melanoma and in control patients, a total of 53 variants were found. Despite this, previously published POT1 variants described in melanoma pedigrees (robles-espinoza *et al.* 2014; shi *et al.* 2014) were not detected in our study. However, we found the intronic variant, g.124467236 T>C, in a patient with a positive family history of melanoma which was described in a patient with multiple primary melanomas carrying the variant previously (shi *et al.* 2014). The region of the variant g.124467236 T>C is highly conserved and according to in silico analyses, this variant possibly causes a change in the splice site. Taken together, this finding supports the idea that this variant is associated with melanoma (shi *et al.* 2014). Our case with the g.124467236 T>C germline variant in POT1 harbored a non-effective variant in CDKN2A at the position g. 21971211 G>C (c.151-4 G>GC) (burgstaller-muehlbacher *et al.* 2015). As described previously, no effect on splicing could be confirmed when the transcript was analyzed (burgstaller-muehlbacher *et al.* 2015).

Of the 53 genetic variants found, 8 were exclusive in high-risk melanoma patients. Two of them, g.124491977 T>G and g.124510982 G>A, both non-synonymous variants, were tested for their alleged functionality. While g.124491977 T>G was predicted to be neutral by all 10 tools, g.124510982 G>A was predicted to be damaging by 80% of the prediction tools and is therefore very likely to be biologically functional. Comparing the wild type amino acid arginine with the resulting cysteine, there are differences in some amino acid features. The mutant residue is smaller and charged neutral, compared to the negatively charged wild type amino acid. Consequently, the correct folding of the protein could be influenced due to the more hydrophobic nature of the resulting amino acid (venselaar *et al.* 2010).

One potential limitation of this study is the fact that family history was largely reported and histopathologic reports confirming the diagnosis of relatives were not available for all cases. In the current study, the potential effect of the variants was assessed by computational analyses. Naturally, functional analyses are required to determine the exact role of these variants in melanoma development.

In conclusion, melanoma driving POT1 germline variants might be rare. However, further studies are required to assemble comprehensive information on the frequency and the role of POT1 in familial melanoma. It is also important to note that germline variants in POT1 were reported to be associated with other types of cancer such as colorectal cancer (chubb *et al.* 2016), glioma (bainbridge *et al.* 2015) and chronic lymphatic lymphoma (calvete *et al.* 2015; karami *et al.* 2016; speedy *et al.* 2016). As none of the variants described were found in melanoma cases, further studies might reveal that POT1 variants are specific to specific cancer types.
